# Trajectories of Canadian Workers’ Well-Being During the Onset of the COVID-19 Pandemic

**DOI:** 10.1007/s11482-024-10397-8

**Published:** 2025-01-07

**Authors:** Tyler Pacheco, Simon Coulombe, Nancy L. Kocovski

**Affiliations:** 1 Department of Industrial Relations, Université Laval, Québec City, QC Canada; 2 Member of the Relief Research Chair in Mental Health, Self-management, and Work, Université Laval, Québec City, QC Canada; 3 Relief Research Chair in Mental Health, Self-management, and Work, Université Laval, Québec City, QC Canada; 4 VITAM – Sustainable Health Research Centre, Québec City, QC Canada; 5 CERVO Brain Research Centre, Québec City, QC Canada; 6 Centre d’études et d’interventions en santé mentale, Université Laval Québec City, Québec City, QC Canada; 7 Centre for the Study of Democratic Citizenship, Montréal, QC Canada; 8 Department of Psychology, Wilfrid Laurier University, Waterloo, ON Canada

**Keywords:** COVID-19, Well-being, Workers, Latent trajectory analysis, Canada

## Abstract

Research regarding workers’ well-being over time during COVID-19 has primarily used variable-centered approaches (e.g., ANOVA) to explore changes in negative well-being. However, variable-centered approaches provide insufficient information on the different well-being experiences that diverse workers may have experienced during COVID-19. Furthermore, researchers have understudied positive well-being in workers’ general lives and work during COVID-19. We used latent trajectory analysis, a person-centered analysis, to explore diverse well-being trajectories Canadian workers experienced during the first few months of COVID-19 across distress, flourishing, presenteeism, and thriving at work measures. We hypothesized that: H1) Intragroup differences would be present on each well-being indicator at study onset; H2) Different longitudinal trajectories would emerge for each well-being indicator (i.e., some workers’ scores would get better, some would get worse, and some would remain the same); and H3) Factors at different ecological levels (self, social, workplace, pandemic) would predict membership to the different trajectories. Canadian workers (*N* = 648) were surveyed March 20-27th, April 3rd-10th, and May 20-27th of 2020. Depending on the well-being indicator, and supporting H1, three to five well-being trajectories were identified. Providing some support for H2, distress and presenteeism trajectories improved over time or stayed stagnant; flourishing and thriving at work trajectories worsened or stayed stagnant. Providing some support for H3, self- (gender, age, disability status, trait resilience), social- (family functioning), workplace- (employment status, financial strain, sense of job security), and pandemic-related (perceived vulnerability to COVID-19) factors significantly predicted well-being trajectory membership. Recommendations for diverse stakeholders (e.g., employers, mental health organizations) are discussed.

The beginning of the coronavirus (COVID-19) pandemic was a turbulent period worldwide, characterized by periods of widespread pathogen, death, as well as social distancing policies (e.g., Dong et al., 2020; World Health Organization, n.d.). Additionally, the pandemic caused several negative work outcomes (e.g., layoffs and closures; Bartik et al., 2020). The compounding of these stressors made a unique set of circumstances that affected workers’ well-being. In a recent study, we show the benefit of exploring both positive and negative well-being in workers’ general lives and at work, as well as the benefit of using person-centered analyses to explore well-being. Canadian workers were found to be experiencing one of five well-being realities (moderately prospering, prospering, moderately suffering, suffering, mixed) early during COVID-19 (Pacheco et al., 2024). Although these results showed that Canadian workers’ well-being during COVID-19 cannot be boiled down to a singular experience, the findings were cross-sectional. Here, we explore the diverse well-being trajectories Canadian workers experienced across time during the first few months of the pandemic.

## Worker Well-Being During COVID-19

Although there is no formal consensus on conceptualizations of well-being, they tend to be either pathogenic or salutogenic. The pathogenic approach is more dominant and views well-being as the absence of disability, disease, and premature death (Keyes & Michalec, 2009). The salutogenic approach is more contemporary and views well-being as “the presence of positive states of human capacities and functioning in cognition, affect, and behavior” (Keyes, 2014, p. 179). Posited by the Two Continua Model (e.g., Westerhof & Keyes, 2010), combining these distinct but related dimensions of well-being provides a more complete perspective that would not otherwise be obtained by solely investigating the individual dimensions. Exploring workers’ complete well-being would provide an unparalleled perspective into workers’ experiences, but researchers have primarily highlighted how COVID-19 has led to workers experiencing negative well-being in general and at work (e.g., Benfante et al., 2020; Choi et al., 2020; Shi et al., 2022). This leaves a paucity of research exploring positive well-being in times of COVID-19 (Waters et al., 2022). As negative and positive well-being are separate but correlated dimensions of well-being (Keyes, 2005), the prioritization of research on negative well-being has resulted in researchers understanding only how some parts of workers’ holistic well-being is affected during COVID-19. As there has also been an increased focus on evidence-based psychological interventions (e.g., see Barlow et al., 2013; Cook et al., 2017), practitioners who aim to work from a well-being promotion perspective do not have much data on how to do so.

Many researchers have explored workers’ negative well-being longitudinally in pandemic-related contexts. Li et al. (2020) found that physicians in China experienced a decline in mood, and more fear of violence and mental health symptoms after the outbreak of COVID-19. In Canada, 77% of healthcare workers who reported working with patients with suspected or confirmed infection reported worsening mental health after the start of COVID-19 (Statistics Canada, 2021). Similarly, healthcare workers in Singapore had a mild increase in perceived stress during COVID-19 (March-August 2020) (Teo et al., 2021). In contrast, and highlighting some workers’ resilience to aversive events, Cyr et al. (2022) found that healthcare workers in Québec, Canada, had no well-being deterioration over the first year of COVID-19. Burnout and anxiety were stable over the year; depression and post-traumatic stress decreased. As these correlated well-being indicators appear to be differentially affected, these findings highlight the benefit of exploring workers’ well-being across multiple negative well-being indicators.

Less is known about the longitudinal changes in workers’ positive well-being during COVID-19. During the lockdown period in Spain, adults’ presence, and search of meaning in life declined (Baños et al., 2023). This decline was found to plateau when strict lockdown measures began to soften. Emerging adults in Germany had significantly lower life satisfaction during COVID-19 than before the pandemic (Preetz et al., 2021). Between 2017 and 2021, positive emotions were twice as frequent as negative ones. The average frequency was the same during COVID-19 (2020-21) (Helliwell et al., 2022). As seen across these studies, it is unclear whether positive well-being is negatively affected during COVID-19, thus highlighting the need for more research on positive well-being outcomes.

## Work-related Well-Being

Positive and negative well-being should also be explored in a work context. Those that adopt the pathogenic perspective may view well-being at work as experiences of presenteeism (i.e., workers being at work but not fully functioning due to illness or other medical conditions; Hemp, 2004); those that adopt the salutogenic perspective may view well-being as experiencing thriving (i.e., a sense of vitality and learning) at work (e.g., Kleine et al., 2019).

Workers’ burnout, as a form of negative work-related well-being, was frequently studied over COVID-19. Peccoralo et al. (2023) reported that frontline healthcare workers in New York City experienced a 5.90% increase in burnout between April-May 2020 (38.90%) and November-January 2021 (44.80%). Müller et al. (2023) found that healthcare worker burnout increased internationally between May-October 2020 and February-April 2021. Other important constructs, such as presenteeism, however, remain largely underexplored. In non-COVID-19-related circumstances, Bryan et al. (2022) reported that there is a predicted threefold increase in the average probability of dysfunctional presenteeism (e.g., 6–18%) when moving between good and poor mental health.

Regarding positive well-being at work, Cotofan et al. (2021) reported that initial decreases in US workers’ workplace happiness were seen in the beginning of the COVID-19 crisis (January-February 2020). This decrease in happiness plateaued about when the national state of emergency was declared and social distancing policies were instated. In March and April, workplace happiness increased. Shortly after, a general downward trend was observed as workplace happiness decreased until January 2021(Cotofan et al., 2021). Highlighting the malleability of work-related well-being and resilience, Cotofan et al. (2021, p. 175) stated, “resilience ostensibly began to wear off and the long-haul nature of the pandemic became a reality.” However, the well-being of all workers likely was not affected equally, and it could have depended on multiple factors.

## Factors Impacting Workers’ Well-Being

Factors can protect individuals’ well-being or put it at risk. Resilience factors protect individuals against the effects of stressors; risk factors increase vulnerability to stressors (Diehl et al., 2012). Inspired by Ecological System Theory (Bronfenbrenner, 1986), we recently showed in a scoping review that several commonly explored factors at self- (age, gender), social- (social support), workplace- (occupation, frontline status), and pandemic-related (risk/exposure, knowing someone infected/killed by the virus, personal protective equipment access) ecological levels were related to worker well-being during such crises (Pacheco et al., submitted). In an empirical study using data collected during May of 2020, we identified five well-being realities (moderately prospering, prospering, moderately suffering, suffering, and mixed) that Canadian workers experienced during COVID-19 (Pacheco et al., 2024). Like the scoping review, factors at self- (gender, age, trait resilience, disability status), social- (marital status, having children at home, family functioning), workplace- (some employment statuses, some work industries, financial strain, job security), and pandemic-related (perceived vulnerability to COVID-19, social distancing) ecological levels predicted membership to diverse well-being realities. As these findings were cross-sectional, the current study aimed to identify factors related to different longitudinal well-being trajectories during the onset of COVID-19.

Acknowledging that diverse factors in workers’ lives are related to well-being is consistent with the third wave of positive psychology. According to Wissing (2022), the third wave adopts a multi-, inter-, or transdisciplinary approach to study well-being. Given the complexity of COVID-19, one could advantageously use this approach to better understand well-being during the pandemic. Reflecting this approach, we marry perspectives rooted in multiple disciplines: industrial-organizational psychology and industrial relations (e.g., precarious work, Allan et al., 2021), developmental and community psychology (e.g., Ecological Systems Theory, Bronfenbrenner, 1986; Jason et al., 2016), positive psychology (Resilience Theory, Pan & Chan, 2007; The Two Continua Model, Keyes, 2005; Westerhof & Keyes, 2010), and public health (social determinants of mental health; Alegría et al., 2018) to better understand workers’ well-being during COVID-19.

## Well-Being Trajectories, Resilience, and Person-centered Analyses

Variable-centered approaches (e.g., analysis of variance, correlation, regression) are parsimonious but lack richness (Howard & Hoffman, 2018). They use data from many participants, and a common trend is identified for the whole sample (Howard & Hoffman, 2018). However, workers can experience a diverse range of well-being realities in (non-)pandemic contexts. Extending from Resilience Theory (Pan & Chan, 2007) and the social determinants of mental health (Alegría et al., 2018), workers could adapt (i.e., bounce back) to COVID-19-related stressors differently depending on which factors are present in their individual and social lives. Thus, using variable-centered approaches may oversimplify these otherwise complex well-being trajectories. In contrast, person-centered analyses (PCA), such as latent trajectory analysis (LTA), can answer important theoretical questions regarding how many well-being trajectories are occurring and who is prospering versus suffering or having mixed experiences. Such research, more broadly, has particular importance in studying and improving quality of life. Extending from He et al.’s (2024) perspective that PCA can aid in developing and facilitating tailored personalized strategies for renal disease symptom management, LTA could be useful to help create personalized strategies geared towards improving poorer well-being trajectories that some groups of workers experienced during and after the pandemic. Such personalized strategies may also be implemented at the onset of future crises to ensure the stability of workers’ quality of life and well-being during these times. Here, LTA is beneficial as it allows for the exploration of intraindividual change and interindividual heterogeneity across multiple timepoints (Meadows et al., 2006).

The benefit of using LTA in such contexts has been demonstrated in a very limited number of studies exploring well-being during COVID-19 (e.g., Jankowski et al., 2023; Shahar et al., 2022). Shahar et al. (2022) identified multiple latent trajectories of Jewish-Israeli adults using general and virus-specific anxiety-related instruments. Whereas three latent trajectories (threat-sensitivity, panic, complacency) were present for both measures of anxiety, a fourth (balanced) was discovered for only general anxiety. Although these findings show how researchers can use LTA to explore well-being trajectories through time, anxiety was the only well-being indicator explored. In addition to only exploring one single negative well-being indicator, well-being was only explored generally (i.e., not considering work as a life domain). The current study aims to address these limitations and explores the multiple longitudinal realities Canadian workers experienced during the pandemic in terms of their positive and negative well-being in their general lives and at work.

## The Present Study

Using LTA, this study has two research questions:

1. What well-being “realities” are experienced by Canadian workers through time across several indicators (distress, flourishing, presenteeism, thriving at work)?
2. What factors at different ecological levels predict membership to the identified well-being trajectories?

Using LTA and inspired by Resilience Theory (Pan & Chan, 2007), social determinants of mental health (Alegría et al., 2018), as well as our previous scoping review and cross-sectional empirical findings (Pacheco et al., submitted, 2024), we tested three hypotheses in the current study. We predicted that: H1) Intragroup differences would be present in levels of each well-being indicator at study onset; H2) Different trajectories would emerge for each well-being indicator, such that some workers’ scores would get better, some workers’ scores would get worse, and some workers’ scores would remain the same through time; and H3) Multiple factors found at different ecological levels (self, social, workplace, pandemic) would predict well-being trajectories’ membership. The hypothesized directions of each factor’s relationship to workers’ well-being are presented in Table 1.

## Methods

### Participants

**Table 1:** Hypothesized Relationships Between the Explored Factors and Well-being Trajectories

Constructs	Relatively better trajectory	Relatively worse trajectory
**Self-related factors**		
Gender		
Men	✓	
Women		✓
Higher age	✓	
Has a disability		✓
Higher trait resilience	✓	
**Social-related factors**		
Higher family functioning	✓	
Children at home		
None		✓
One or more	✓	
**Workplace-related factors**		
Employment status		
Employed full-time	✓	
Employed part-time Laid off due to COVID-19 Other unemployed (e.g., not employed but looking)		✓ ✓ ✓
Higher job security	✓	
Higher financial strain		✓
**Pandemic-related factors**		
Higher perceived vulnerability to COVID-19		✓
Social distancing		
No	✓	
Yes		✓

Participants had to be 18 years old, living in Canada, working at least 20 h per week before COVID-19, and able to read English. Data were deleted at each timepoint if participants failed more than one of the three attention checks (e.g., “Please make sure to select ‘A somewhat positive change’ for this answer so that we know you are paying attention”) embedded in each survey. Data were also missing at each timepoint due to attrition. Looking at the full dataset, across the three timepoints, there were 1,355 distinct participants. Time 0 had 1,201 workers (including seven that failed attention checks), Time 1 had 666 workers (including six that failed attention checks), and Time 2 had 516 workers (including two that failed attention checks).^1^

After removing participants’ data at each timepoint for failing more than one attention check, 700 participants completed one timepoint, 276 completed two timepoints, and 372 completed all three timepoints, the latter two comprise the final sample used for this study (*N* = 648). It is important to note that preliminary attrition analyses suggested that participants with only one completed timepoint had worse well-being (see “Descriptive Statistics and Preliminary Analyses” results section). As we aimed to explore the longitudinal well-being trajectories Canadian workers were experiencing, we only included workers with two or three completed timepoints. Table 2 contains the demographic information of the full sample of workers whose data were retained for our final analyses, as well as a breakdown by sampling source (social media, Qualtrics). As seen in Table 2 (and similar to the proportions in Pacheco et al., 2024), the social media subsample had a larger proportion (%) of those who had no children at home. The social media subsample also had a larger proportion (%) of those who were: women or a gender minority, single, in a common law or other romantic relationship, White/Caucasian, living in New Brunswick, Ontario, or Yukon, born in Canada, or disabled. The social media subsample also had a larger proportion (%) of those who (were): employed part-time, temporarily or indefinitely laid off, in the food, healthcare, or services industry, scheduled to work zero to less than 30 h of work a week or 30 to less than 40 h a week. The social media subsample had younger workers and workers with higher financial strain. The Qualtrics subsample had a larger proportion (%) of those who had one or more children at home. The Qualtrics subsample also had a larger proportion (%) of those who were: men, married, separated, divorced, widowed, a racial minority, living in Alberta, British Columbia, Manitoba, Newfoundland and Labrador, Northwest Territories, Prince Edward Island, Québec, or Saskatchewan, an immigrant, or not disabled. The Qualtrics subsample also had a larger proportion (%) who were: employed full-time, working in a manufacturing or “other” (e.g., automotive, construction) industry, or scheduled to work 40 h or more. The Qualtrics sample had older workers and workers with lower financial strain. Whereas differences were found in the proportion of workers in each subsample living in different provinces and territories, the proportion of those living in Nova Scotia and Nunavut were the same in both subsamples. The effect sizes were mainly small across the demographic-related variables. However, the effect size was medium for gender and large for age.

**Table 2:** Sample Characteristics

Variables	Total (*N* = 648)	Social Media (*n* = 276)	Qualtrics (*n* = 372)	t^d^or χ^2e^Statistic	Effect Size^f^
	Frequency (%) or Mean (SD)	Frequency (%) or Mean (SD)	Frequency (%) or Mean (SD)		
**Age (in Years)** ^g^	41.45(12.23)	34.30(10.03)	46.31(11.16)	-14.04*** ^*d*^	-1.12
**Gender**				143.74*** ^*e*^	0.48
Men Women Other Missing/Prefer not to say (%)	209(32.30) 406(62.70) 9(1.40) 3.70	16(5.80) 228(82.60) 8(2.90) 8.70	193(51.90) 178(47.80) 1(0.30) 0.00		
**Marital Status**				51.91*** ^*e*^	0.29
Single Common law Married Separated Divorced Widowed Other Missing (%)	189(29.20) 92(14.20) 264(40.70) 17(2.60) 29(4.50) 10(1.50) 23(3.50) 3.70	101(36.60) 44(15.90) 69(25.00) 7(2.50) 11(4.00) 3(1.10) 18(6.50) 8.30	88(23.70) 48(12.90) 195(52.40) 10(2.70) 18(4.80) 7(1.90) 5(1.30) 0.30		
**Number of Dependents in Household**				12.96*** ^*e*^	0.15
None One or more Missing (%)	442(68.20) 173(26.70) 5.10	195(70.70) 49(17.80) 11.60	247(66.40) 124(33.30) 0.30		
**Race**				6.54** ^*e*^	0.10
Caucasian/White Racial minority Missing/Prefer not to say (%)	542(83.60) 71(11.00) 5.40	231(83.70) 19(6.90) 9.40	311(83.60) 52(14.00) 2.40		
**Residing Province or Territory**				28.66** ^*e*^	0.21
Alberta British Columbia Manitoba New Brunswick Newfoundland and Labrador Northwest Territories Nova Scotia Nunavut Ontario Prince Edward Island Québec Saskatchewan Yukon Missing (%)	66(10.20) 88(13.60) 26(4.00) 28(4.30) 11(1.70) 1(0.20) 42(6.50) 0(0.00) 293(45.20) 1(0.20) 40(6.20) 27(4.20) 2(0.30) 3.50	25(9.10) 35(12.70) 8(2.90) 14(5.10) 4(1.40) 0(0.00) 18(6.50) 0(0.00) 136(49.30) 0(0.00) 9(3.30) 2(0.70) 2(0.70) 8.30	41(11.00) 53(14.20) 18(4.80) 14(3.80) 7(1.90) 1(0.30) 24(6.50) 0(0.00) 157(42.20) 1(0.30) 31(8.30) 25(6.70) 0(0.00) 0.00		
**Born in Canada**				15.70*** ^*e*^	0.16
Yes No Missing (%)	518(79.90) 107(16.50) 3.50	228(82.60) 25(9.10) 8.30	290(78.00) 82(22.00) 0.00		
**Has One or More Disabilities**				52.16*** ^*e*^	0.29
Yes No Missing/Prefer not to say (%)	94(14.50) 521(80.40) 5.10	69(25.00) 176(63.80) 11.20	25(6.70) 345(92.70) 0.50		
**Financial Strain** ^g^	2.17(0.92)	2.36(0.95)	2.03(0.88)	4.41*** ^*d*^	0.36
**Employment Status** ^b^					
Employed full-time Employed part-time On Mental health/addiction leave Retired Not employed but looking Not employed nor looking Receiving disability support Receiving social assistance Temporarily laid off Indefinitely laid off Missing (%)	447(69.00) 53(8.20) 2(0.30) 0(0.00) 13(2.00) 7(1.10) 1(0.20) 2(0.30) 108(16.70) 15(2.30) 3.50	148(53.60) 33(12.00) 2(0.70) 0(0.00) 7(2.50) 5(1.80) 1(0.40) 1(0.40) 62(22.50) 10(3.60) 8.30	299(80.40) 20(5.40) 0(0.00) 0(0.00) 6(1.60) 2(0.50) 0(0.00) 1(0.30) 46(12.40) 5(1.30) 0.30	36.14*** 11.33*** 2.94 - 0.97 2.80 1.47 0.07 15.41*** 4.35*	0.24 0.13 - - - - - - 0.16 0.08
**Industry of Work** ^a b^					
Education Food Healthcare Manufacturing Services Other (e.g., automotive, construction) ^c^ Missing (%)	87(13.40) 43(6.60) 102(15.70) 42(6.50) 88(13.60) 301(46.50) 3.50	42(15.20) 28(10.10) 53(19.20) 5(1.80) 46(16.70) 109(39.50) 8.30	45(12.10) 15(4.00) 49(13.20) 37(9.90) 42(11.30) 192(51.60) 0.00	2.55 11.63*** 6.67** 15.26*** 5.91* 4.39*	- 0.14 0.10 0.16 0.10 0.08
**Telecommuting Off-site** ^a^				0.33	-
Yes No Missing (%)	263(40.60) 362(55.90) 3.50	103(37.30) 150(54.30) 8.30	160(43.00) 212(57.00) 0.00		
**Average Hours of Scheduled Work per Week** ^a^				21.45*** ^*e*^	0.19
0 to less than 30 h 30 h to less than 40 h 40 h or more Missing (%)	197(30.40) 214(33.00) 210(32.40) 4.20	98(35.50) 94(34.10) 59(21.40) 9.10	99(26.60) 120(32.30) 151(40.60) 0.50		
**Socially Distancing**				0.15 ^*e*^	-
Yes No Missing (%)	547(84.40) 78(12.00) 3.50	223(80.80) 30(10.90) 8.30	324(87.10) 48(12.90) 0.00		

*Note*. This demographic breakdown is based on the sample at Time 0. Only participants with more than one completed timepoint are included. ^a^ Frequencies were calculated using only the subsample of participants who reported being employed at the time of the survey. ^b^ Participants were able to select more than one option. ^c^ Industries containing less than 5% of the sample were merged into the “other” category. ^d^ A *t*-test was conducted. ^e^ A chi-squared test was conducted. ^f^*w* is reported for the chi-square statistics; Cohen’s *d* is reported for the *t*-tests. ^g^ Satterthwaite approximation is used due to a significant Levene’s Test. *** *p* ≤ .001, ** *p ≤* .01, * *p* ≤ .05

### Procedure

Wilfrid Laurier University’s Research Ethics Board approved this study (REB #6497). The study had three timepoints. Time 0 was collected between March 20-27th, 2020, one to two weeks after social distancing policies were implemented in Canada. Time 1 was collected between April 3-10th, 2020 (two weeks after Time 0); Time 2 was collected between May 20-27th, 2020 (two months after Time 0).

Participants were recruited via a panel of Canadian workers managed by Qualtrics as well as through social media advertisements. Given concerns regarding the representativeness (i.e., lack of heterogeneity) of online panels (e.g., Scherpenzeel, 2010), both sampling methods were used to obtain a more diverse sample. Regarding advertisements, the researchers posted unpaid study advertisement on their Facebook newsfeeds and several Facebook community groups dedicated to individuals in diverse provinces and territories. Interested users could access more information and/or obtain the survey link from a Facebook page linked to the advertisement. Eligible workers from Qualtrics’ panel accessed the survey directly via hyperlink at each timepoint. Participants consented at each timepoint and answered measures to ensure eligibility at Time 0. At the end of the surveys, social media participants at Time 0 were asked whether they would like to be re-contacted for later surveys. Survey links were e-mailed to those who consented to be re-contacted. For Qualtrics participants, the panel system allowed Qualtrics to re-contact participants for the different timepoints. Social media participants were invited to enter a gift card raffle after each survey; Qualtrics workers received compensation determined by Qualtrics.

### Measures

Table 3 contains the study’s main measures used to (1) identify well-being trajectories experienced by Canadian workers, and (2) predict trajectory membership. Four diverse well-being-related constructs were used to identify well-being trajectories experienced by Canadian workers at the beginning of COVID-19: distress, flourishing, presenteeism, and thriving at work. Aside from sociodemographic-related variables, factors at distinct ecological levels (self, social, workplace, pandemic) were selected to test if they would predict membership to the well-being trajectories. The multi-item measures used in the study had good to excellent internal reliabilities across the timepoints in this study (see Table 3 for Cronbach’s alphas).

### Data Analysis

SPSS (IBM Corp., 2021, V. 28.0) was used for data preparation and conducting descriptive and preliminary analyses. MPlus (Muthén & Muthén, 1998–2017, V. 8.4) was used to address the study’s main objectives.

As some of the well-being indicators investigated by Pacheco et al. (2024) were only moderately correlated, we conducted LTA for each individual well-being indicator. Rather than modelling these well-being indicators together, where they would be considered simultaneously, individual exploration of these constructs allowed us to see the different non-linear well-being trajectories workers were experiencing on each indicator. To test hypotheses 1 and 2 (intragroup differences in well-being at study onset and the presence of diverse well-being trajectories, respectively), LTA was conducted seven times (each model including from 1 to 7 trajectories) for each well-being indicator, with each subsequent model containing an additional trajectory. This modelling was conducted using the Robust Maximum Likelihood (MLR) estimator. The MLR estimator was used as it is robust to non-normality and missing data when conducting latent growth curve analyses, as well as yields more accurate standard errors and growth parameter coverage (Shi et al., 2021). Given the slight non-normality in some of our predictors and the attrition present, the MLR estimator was deemed to be the most optimal. Random starts were used during the model-building process to ensure MPlus was replicating the highest log-likelihood (i.e., avoiding local solutions; Berlin et al., 2014). Following Morin’s (2016) recommendation, we used 3,000 sets of random starts, 100 iterations of these random starts, and 100 of these starts for final stage optimization. Each of the model’s outputs were inspected to ensure that the best log-likelihood was replicated at least five times. The model selection process in the following steps were inspired by Ram and Grimm (2009) (also see Spurk et al., 2020). First, the AIC, BIC, SSA-BIC, and CAIC were reviewed for each model. Lower values in these relative indices of fit indicated better fit (Ferguson et al., 2020; Masyn, 2013). Second, the VLMR, ALMR, and BLRT were investigated for each model. A significant ratio test indicated that the model with *k* trajectories was significantly better than one with *k* minus one trajectory. Third, the entropy was evaluated for each model. An acceptable entropy is 0.80 or more (Weller, 2020). However, the overall pattern of the indices of fit and ratio tests in steps 1 and 2 were considered along the recommendations regarding the model’s entropy. Lastly, the models’ outputs were reviewed for errors (e.g., out-of-bound parameters, theoretical plausibilities). This step represented an ongoing process that was checked after conducting each model. No concerns were present in the model outputs. Previous studies have also followed an additional recommendation that models containing clusters with less than 5% of the sample should not be retained (Hamza & Willoughby, 2013). Given the complexity of well-being through time during pandemics, and like other researchers who showed the benefits of reporting on smaller unique latent profiles (e.g., Nguyen & Stinglhamber, 2020; Park et al., 2022), we did not follow this recommendation here. We did this in the hope that smaller yet diverse and theoretically significant trajectories would emerge. Implementing this recommendation could have meant not retaining a model with unique well-being trajectories that were otherwise statistically better.

Hypothesis 3 (ecological factors predicting well-being trajectories’ membership), once the number of well-being trajectories were identified, was tested using measures answered at Time 0. Time 0 survey items optimally predict trajectory membership as they reflect the factors workers had in their lives closer to the pandemic onset. Although participants without Time 0 data were retained in our analyses, their responses could not be used to test hypothesis 3 because they had no data at Time 0. The hypothesized predictors of trajectory membership were tested using Mplus’ auxiliary functions, added to the LTA syntax. To do so, in cases of a continuous variable (i.e., predictor), using the BCH function (Bakk & Vermunt, 2016), their means were individually compared across the latent trajectories (Asparouhov & Muthén, 2021). In the case of a categorical variable, using the DCAT function (Lanza et al., 2013), we tested whether the distributions of participants across the categories of the variable were the same between the latent trajectories (also see: Reinhardt et al., 2022). Multiple predictors were tested, however, and some variables had categories with a small proportion (< 5%) of the sampled workers. With such small categories, issues of power could have arisen especially when testing their predictive associations with well-being trajectories that are less frequent. Thus, categories containing less than 5% of the sample were either (a) removed from these analyses or (b) merged with other categories. Merging categories together only occurred when it made conceptual sense to do so (e.g., laid off temporarily and laid off indefinitely were merged, but widowed and “other” romantic partner were not merged).

**Table 3:** Main Study Measures

Construct	Scale/Item	# of Items	Scale Range	α
**Trajectory Indicators**				
Distress	Patient Health Questionnaire-4 (Löwe et al., 2010)	4	Not at all (1); Nearly every day (4)	0.90-0.92
Flourishing	The Psychological Well-being Scale (Diener et al., 2009)	8	Strongly disagree (1); Strongly agree (7)	0.91-0.93
Presenteeism	The Employment Absence and Productivity Scale (Lam et al., 2009)	7	None of the time (0%) (1); All the time (100%) (5)	0.91-0.92
Thriving at work	Index of Psychological Well-Being at Work (Dagenais-Desmarais & Savoie, 2012)	5	Disagree (0); Completely agree (5)	0.95
**Potential Predictors of Trajectory Membership**				
Family functioning	Family APGAR Scale (Smilkstein et al., 1982)	5	Hardly ever (1); Almost always (3)	0.90-0.92
Financial strain	“How would you describe the money situation in your household right now?” (Huntley et al., 1993; Okechukwu et al., 2012; Szanton et al., 2008)	1	Comfortable with extra (1); Cannot make ends meet (4)	-
Perceived Vulnerability to COVID-19	Perceived Susceptibility Scale (Yoo et al., 2016)	3	Strongly disagree (1); Strongly agree (5)	0.91-0.96
Social distancing	“Have you isolated yourself from others (i.e., social distancing) to prevent contaminating others or being contaminated with the coronavirus (COVID-19)?”	1	No (1); Yes (2)	-
Sense of Job Security	Crisis and/or Disaster Preparedness Scale (Fowler et al., 2007)	7	Strongly disagree (1); Strongly agree (7)	0.86-0.87
Trait Resilience	Brief Resilience Scale (Smith et al., 2008)	3	Strongly disagree (1); Strongly agree (5)	0.83-0.87

*Note.* α = Cronbach’s alpha range across the three timepoints. For Thriving at work, alpha was the same at all timepoints

## Results

### Descriptive Statistics and Preliminary Analyses

Table 4 contains the descriptive statistics and bivariate correlations for the well-being indicators over time. Using all the valid cases across the three timepoints, there were some missing data for the measures of distress and flourishing (3.50–28.40%). There was more missing data on presenteeism and thriving at work (5.51-40.00%). Using an absolute value of 1 (e.g., see Ramos et al., 2018), most of the well-being indicators at every timepoint were normally distributed, with three exceptions. Flourishing at Time 0 was kurtotic, presenteeism at Time 1 was skewed, and presenteeism at Time 3 was skewed and kurtotic. Q-Q plot inspection showed that some well-being measures were not normal. Irrespective of this non-normality, the MLR estimator is robust to such deviations (Shi et al., 2021).

**Table 4:** Descriptive Statistics and Bivariate Correlations for the Well-being Indicators

Construct	*N*	*M*	*SD*	Skewness	Kurtosis	1.	2.	3.	4.	5.	6.	7.	8.	9.	10.	11.	12.
**Time 0**																	
1. Distress	625	2.27	0.95	0.40	-0.98	-											
2. Flourishing	625	5.34	1.05	-0.96	1.34	− 0.43	-										
3. Presenteeism ^a^	498	2.02	0.89	0.85	0.15	0.68	− 0.42	-									
4. Thriving at Work ^a^	600	4.31	1.39	-0.70	-0.36	− 0.24	0.40	− 0.31	-								
**Time 1**																	
5. Distress	576	2.13	0.90	0.61	-0.66	0.73	− 0.40	0.59	− 0.23	-							
6. Flourishing	575	5.27	1.04	-0.63	0.30	− 0.41	0.73	− 0.39	0.34	− 0.50	-						
7. Presenteeism ^a^	455	2.01	0.87	1.02	0.71	0.56	− 0.43	0.67	− 0.30	0.69	− 0.49	-					
8. Thriving at Work ^a^	543	4.19	1.35	-0.69	-0.24	− 0.26	0.34	− 0.28	0.80	− 0.30	0.39	− 0.33	-				
**Time 2**																	
9. Distress	465	1.90	0.89	0.81	-0.38	0.73	− 0.45	0.62	− 0.30	0.72	− 0.48	0.63	− 0.34	-			
10. Flourishing	464	5.24	1.11	-0.73	0.20	− 0.48	0.74	− 0.41	0.37	− 0.45	0.71	− 0.44	0.39	− 0.61	-		
11. Presenteeism ^a^	380	1.84	0.80	1.18	1.21	0.61	− 0.43	0.69	− 0.30	0.65	− 0.43	0.74	− 0.29	0.74	− 0.55	-	
12. Thriving at Work ^a^	381	4.25	1.35	-0.77	-0.06	− 0.36	0.41	− 0.38	0.79	− 0.29	0.47	− 0.34	0.77	− 0.44	0.53	− 0.41	-

*Note.*^a^ Construct only measured among those with current or recent employment at the time of the survey. All correlations were significant at *p* < .001

Attrition was tested by correlating workers’ well-being scores at Time 2 with the number of valid timepoints. Results indicated that workers with better scores on the well-being indicators at Time 2 had more valid completed timepoints (distress: *r*[509] = − 0.15, *p* < .001; flourishing: *r*[508] = 0.14, *p* = .002; presenteeism: *r*[411] = − 0.20, *p* < .001; thriving at work: *r*[411] = 0.10, *p* = .04).

### Model Selection

**Fig. 1:**
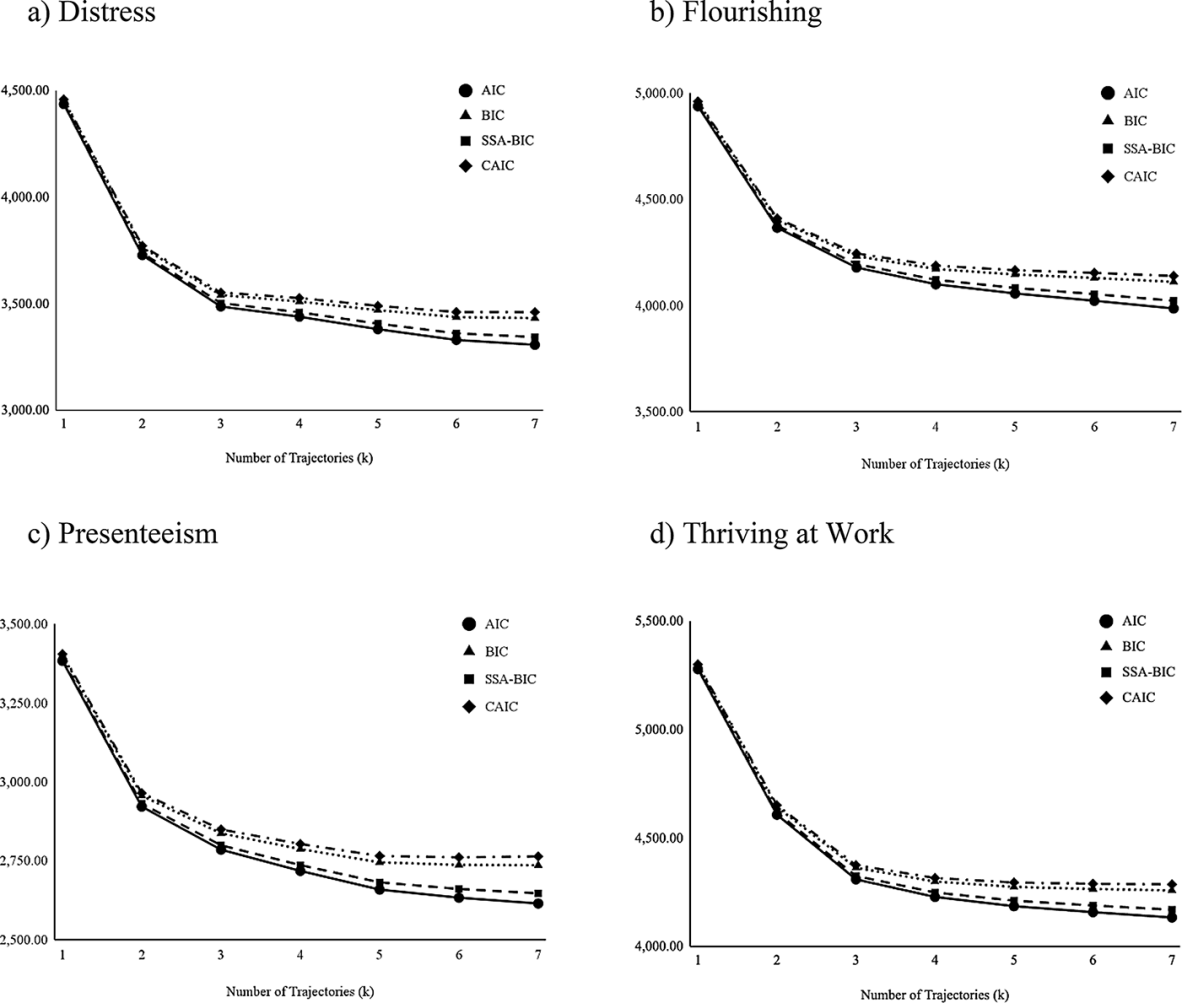
Graphs of Indices of Fit for Each Well-being Indicator with an Increasing Number of Trajectories. **a**) Distress, **b**) Flourishing, **c**) Presenteeism **d**) Thriving at Work

Seven LTA models were tested for each well-being indicator. Table 5 contains the indices of fit for the tested models; Fig. 1 provides a visual representation of the relative improvement across the indices of fit (AIC, BIC, SSA-BIC, CAIC) for each tested model. For each well-being indicator, we first state which model was selected, followed by reasons for rejecting other possible models.

The four-trajectory model for distress was selected. Although the relative indices of fit supported a five- or six-trajectory model, two of the ratio tests (VLMR, ALMR) for both models and entropy for the six-trajectory model suggested that these models did not provide a better fit than the four-trajectory model. For flourishing, the three-trajectory model was selected. Although the seven-trajectory model had significant ratio tests (indicating better fit than a model with one less trajectory) and a sufficient entropy, the relative improvement in the relative indices of fit was marginal. Such a small decrease across these indices from the models with three to seven trajectories suggested that there was no large gain by having more than three trajectories. For presenteeism, the five-trajectory model was selected. Aside from the acceptable entropies, there was no evidence that the six- or seven-trajectory model was a better fit for presenteeism. Lastly, for thriving at work, the four-trajectory model was selected. There was either no support or only mixed support for selecting the models with five, six, or seven trajectories.

**Table 5:** Indices of Fit for the Well-being Models

Number of Trajectories (*k*)	LL	FP	AIC	BIC	SSA-BIC	CAIC	VLMR	ALMR	BLRT	Entropy
**Distress (** ***N*** ** = 648)**										
1	-2,214.49	4	4,436.97	4,454.87	4,442.17	4,458.87	-	-	-	-
2	-1,855.75	8	3,727.50	3,763.30	3,737.90	3,771.30	< 0.001	< 0.001	< 0.001	0.82
3	-1,731.39	12	3,486.78	3,540.46	3,502.37	3,552.46	< 0.001	< 0.001	< 0.001	0.82
**4**	**-1**,**703.39**	**16**	**3**,**438.77**	**3**,**510.36**	**3**,**459.56**	**3**,**526.36**	**0.02**	**0.02**	**< 0.001**	**0.81**
5	-1,670.10	20	3,380.19	3,469.67	3,406.17	3,489.67	0.17	0.18	< 0.001	0.80
6	-1,640.77	24	3,329.55	3,436.92	3,360.72	3,460.92	0.10	0.10	< 0.001	0.79
7	-1,625.43	28	3,306.85	3,432.12	3,343.22	3,460.12	0.45	0.46	< 0.001	0.80
**Flourishing (*N*** **= 648)**										
1	-2,464.95	4	4,937.91	4,955.80	4,943.10	4,959.80	-	-	-	-
2	-2,174.66	8	4,365.31	4,401.11	4,375.71	4,409.11	< 0.001	< 0.001	< 0.001	0.81
**3**	**-2**,**077.11**	**12**	**4**,**178.22**	**4**,**231.90**	**4**,**193.80**	**4**,**243.90**	**0.001**	**0.002**	**< 0.001**	**0.82**
4	-2,033.67	16	4,099.34	4,170.93	4,120.13	4,186.93	0.06	0.06	< 0.001	0.76
5	-2,007.87	20	4,055.74	4,145.22	4,081.72	4,165.22	0.19	0.20	< 0.001	0.80
6	-1,986.73	24	4,021.46	4,128.83	4,052.63	4,152.83	0.14	0.15	< 0.001	0.82
7	-1,964.56	28	3,985.12	4,110.39	4,021.49	4,138.39	0.01	0.01	< 0.001	0.84
**Presenteeism (*N*** **= 558)**										
1	-1,687.59	4	3,383.19	3,400.49	3,387.79	3,404.49	-	-	-	-
2	-1,452.67	8	2,921.33	2,955.93	2,930.53	2,963.93	< 0.001	< 0.001	< 0.001	0.80
3	-1,380.48	12	2,784.96	2,836.86	2,798.76	2,848.86	0.03	0.03	< 0.001	0.80
4	-1,342.84	16	2,717.68	2,786.87	2,736.08	2,802.87	0.003	0.003	< 0.001	0.82
**5**	**-1**,**309.24**	**20**	**2**,**658.47**	**2**,**744.96**	**2**,**681.47**	**2**,**764.96**	**0.05**	**0.05**	**< 0.001**	**0.82**
6	-1,292.40	24	2,632.81	2,736.59	2,660.40	2,760.59	0.15	0.16	< 0.001	0.82
7	-1,279.20	28	2,614.41	2,735.49	2,646.60	2,763.49	0.35	0.36	< 0.001	0.82
**Thriving at Work (*N*** **= 635)**										
1	-2,635.53	4	5,279.06	5,296.88	5,284.18	5,300.88	-	-	-	-
2	-2,295.80	8	4,607.60	4,643.23	4,617.83	4,651.23	< 0.001	< 0.001	< 0.001	0.87
3	-2,142.75	12	4,309.50	4,362.95	4,324.85	4,374.95	< 0.001	< 0.001	< 0.001	0.82
**4**	**-2**,**098.22**	**16**	**4**,**228.43**	**4**,**299.69**	**4**,**248.89**	**4**,**315.69**	**0.02**	**0.02**	**< 0.001**	**0.82**
5	-2,072.86	20	4,185.73	4,274.80	4,211.30	4,294.80	0.10	0.11	< 0.001	0.84
6	-2,055.10	24	4,158.20	4,265.09	4,188.89	4,289.09	0.07	0.08	< 0.001	0.77
7	-2,038.80	28	4,133.61	4,258.31	4,169.41	4,286.31	0.49	0.50	< 0.001	0.80

*Note.* The models with the bolded values were the final well-being models interpreted. LL = loglikelihood; FP = the number of free parameters; AIC = Akaike Information Criteria; BIC = Bayesian Information Criteria; SSA-BIC = Sample-Size Adjusted BIC; CAIC = Consistent AIC; VLMR = Vuong-Lo-Mendell-Rubin Likelihood Ratio Test for k-1 profiles (H_0_) versus k profiles; ALMR = Lo-Mendell-Rubin Adjusted Likelihood Ratio Test for k-1 profiles (H_0_) versus k profiles; BLRT = Parametric Bootstrapped Likelihood Ratio Test for k-1 profiles (H_0_) versus k profiles

Although the entropies of the selected models indicated sufficient confidence that workers were classified in one trajectory than another, we also interpreted the classification probabilities associated with workers being in their respective trajectories. Results indicated that workers across all four models were likelier to be members of their respective trajectories (0.80–0.95) than others (0.00-0.15). In only two cases, correct membership classification was relatively lower than in the other models (i.e., Trajectory 3 of the distress model, 0.66; Trajectory 2 of the presenteeism model, 0.63). Incorrect cross-membership of workers between Trajectory 1 and Trajectory 3 in the distress model (0.25) was also relatively higher than the other comparisons.

### Description of Models

Table 6 contains the trajectories’ coefficients. We use several terms (“lowest,” “lower,” “higher,” “highest”) to represent best subgroups’ well-being than that of the other trajectories. Tables 7, 8, 9 and 10 contain the trajectory membership results. The tables contain all the tested predictors of trajectory membership and their results, but only the significant (*p* ≤ .05) findings are presented hereafter, focusing on the variable categories with the highest proportion of workers or the highest mean scores across the well-being trajectories. For each model, we will first describe the models’ trajectories, considering baseline well-being levels (H1) and the way worker well-being evolves through time (H2). Relevant to H3, we will then present the factors associated with trajectory membership.

**Table 6:** Characteristics of Trajectories for Each Latent Well-being Model

	Intercept Coefficient	Linear Change Coefficient	Quadratic Change Coefficient
*Distress Model*			
Trajectory 1	2.32***	0.07	-0.09
Trajectory 2	1.46***	-0.002	-0.06**
Trajectory 3	3.47***	-1.33**	0.30
Trajectory 4	3.55***	0.14	-0.13
*Flourishing Model*			
Trajectory 1	4.82***	-0.41***	0.16**
Trajectory 2	6.01***	-0.11	0.04
Trajectory 3	3.18***	0.67	-0.38
*Presenteeism Model*			
Trajectory 1	2.16***	0.38	-0.20**
Trajectory 2	3.31***	-2.31**	0.76**
Trajectory 3	3.11***	0.59**	-0.34**
Trajectory 4	1.37***	0.10	-0.06*
Trajectory 5	4.66***	0.07	-0.13
*Thriving at Work Model*			
Trajectory 1	4.28***	-0.20	0.06
Trajectory 2	5.55***	-0.35***	0.13***
Trajectory 3	1.63***	-0.28	0.09
Trajectory 4	2.88***	-0.19	0.09

***p ≤ .001, ** *p* ≤ .01, * *p* ≤ .05

**Fig. 2:**
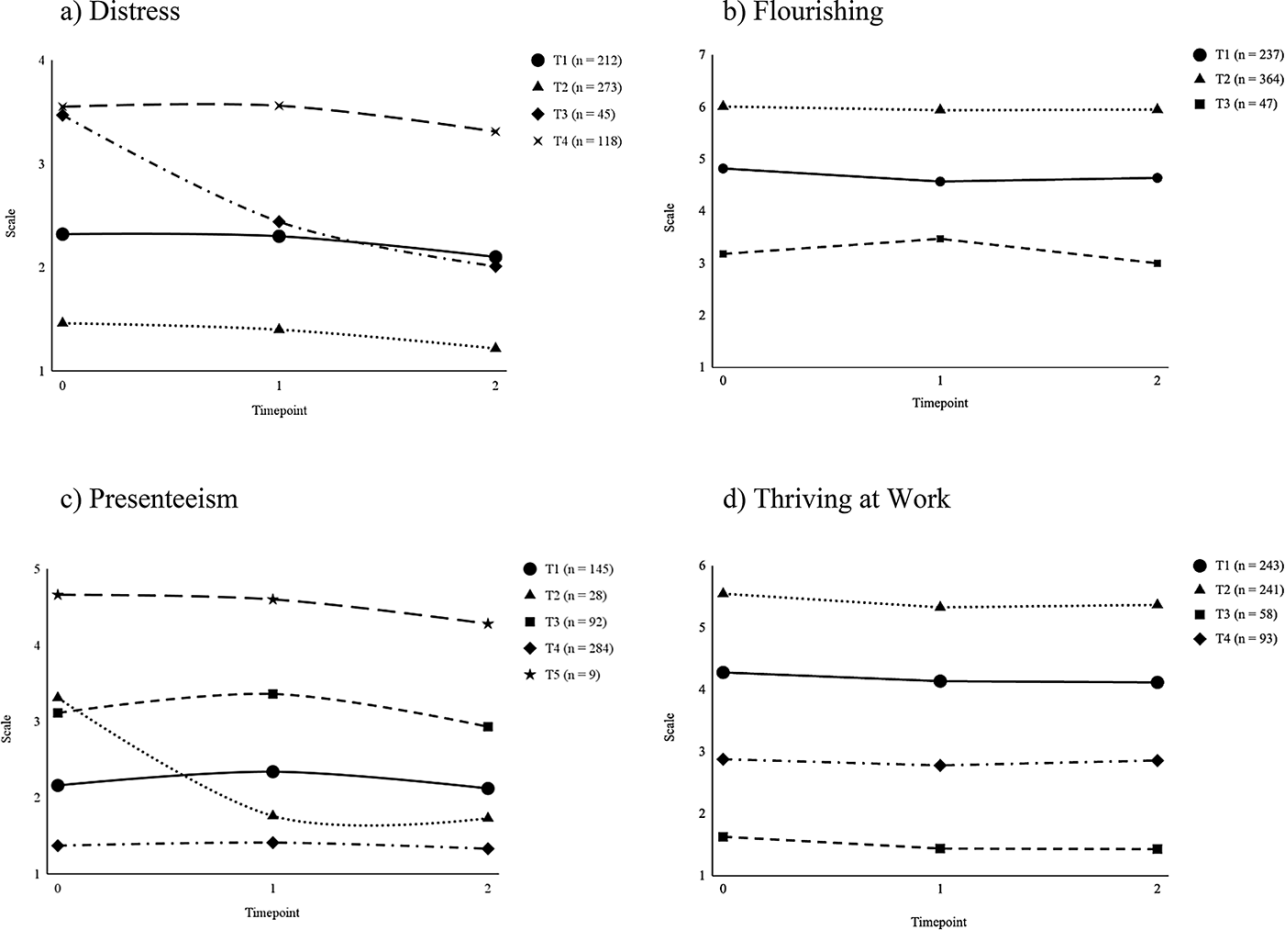
Graphs of the Longitudinal Trajectories for Each Well-being Indicator. (a) Distress (b) Flourishing (c) Presenteeism (d) Thriving at Work

#### Distress Model

The distress model (see Fig. 2a) contained four latent trajectories. Trajectory 1 (32.72% of the sample) had moderate distress at study onset (2.32), which decreased – although not significantly – to 2.10 at Time 2. Trajectory 2 had the most workers (42.13%). These workers had the lowest distress scores overall; they had a low distress score at study onset (1.46), exponentially decreasing to 1.22 at Time 2. Thus, Trajectory 2 was the best trajectory of the four. Trajectory 3 had the least number of workers (6.94%). These workers had higher distress at study onset (3.47), linearly decreasing to 2.01 at Time 2. Trajectory 4 (18.21% of the sample) had the highest distress scores overall; they had a high distress score at study onset (3.55), which decreased – although not significantly – to 3.31 at Time 2. Thus, Trajectory 4 was the worst trajectory of the four.

#### Membership to Latent Distress Trajectories

As seen in Table 7, Trajectory 1 included a larger proportion of racialized and non-telecommuting workers. Trajectory 2 included a larger proportion of workers who: identified as men, were not disabled, worked full-time or scheduled 40 h or more a week. Workers in Trajectory 2 had the highest mean in trait resilience, age, positive family functioning, and sense of job security. Trajectory 2 had a larger proportion of Qualtrics workers. The demographic predictors of Trajectory 2 mirrored the sociodemographic breakdown of our Qualtrics subsample (e.g., mean age is highest in Trajectory 2 and the Qualtrics subsample). Trajectory 3 included a larger proportion of workers who: identified as White/Caucasian, scheduled 30 to less than 40 h a week, were in a form of unemployment unrelated to COVID-19 (e.g., not employed but looking), or were telecommuting. Trajectory 4 included a larger proportion of workers who identified as a woman or had one or more disabilities. Trajectory 4 also had a larger proportion of workers employed part-time, laid off, or scheduled less than 30 h a week. Workers in Trajectory 4 had the highest mean in financial strain and perceived vulnerability to COVID-19. Trajectory 4 had the largest proportion of social media workers. The demographic predictors of Trajectory 4 mirrored the sociodemographic breakdown of our social media subsample.

**Table 7:** Association of Risk and Resilience Factors with Distress Trajectories

	Trajectory 1 Probability^a^ or M (S.E.)^b^	Trajectory 2 Probability^a^or M (S.E.)^b^	Trajectory 3 Probability^a^or M (S.E.)^b^	Trajectory 4 Probability^a^or M (S.E.)^b^	χ^2^
Source Social media Qualtrics	[2,4] 0.57 0.44	[1,3,4] 0.16 0.85	[2] 0.60 0.40	[1,2] 0.69 0.31	146.89***
*Self-related Factors*					
Gender ^a^ Men Women	[2,4] 0.30 0.70	[1,3,4] 0.48 0.52	[2] 0.24 0.76	[1,2] 0.14 0.86	46.45***
Age ^b^	[2,4] 39.03(1.06)	[1,3,4] 46.16(0.78)	[2] 38.81(1.95)	[1,2] 36.00(1.07)	71.00***
Racial minority ^a^	[3,4] 0.17	[3] 0.11	[1,2,4] 0.00	[1,3] 0.09	84.78***
Immigrant ^a^	0.20	0.18	0.16	0.12	4.02
Has one or more disabilities ^a^	[2,4] 0.18	[1,4] 0.08	0.15	[1,2] 0.29	22.54***
Trait resilience ^b^	[2,4] 3.15(0.07)	[1,3,4] 3.86(0.05)	[2,4] 3.02(0.15)	[1,2,3] 2.58(0.10)	188.07***
*Social-related Factors*					
Marital status ^a^					13.30
Single Common law Married Separated/Divorced	0.35 0.18 0.40 0.07	0.28 0.13 0.52 0.08	0.20 0.20 0.50 0.10	0.43 0.15 0.34 0.08	
Family functioning ^b^	[2] 2.41(0.05)	[1,4] 2.61(0.03)	2.38(0.11)	[2] 2.35(0.05)	23.11***
Children at home ^a^	0.29	0.27	0.32	0.27	0.57
*Workplace-related Factors*					
Employment status ^a^		[4]		[2]	26.81**
Employed full-time Employed part-time Laid off due to COVID-19 Other unemployed (e.g., not employed but looking)	0.71 0.08 0.20 0.01	0.81 0.06 0.13 0.01	0.60 0.11 0.22 0.07	0.56 0.13 0.25 0.05	
Work industry ^a^					
Education Food Healthcare Manufacturing Services Other (e.g., automotive)	0.13 0.08 0.18 0.04 0.19 0.50	0.13 0.05 0.17 0.09 0.11 0.49	0.10 0.15 0.18 0.02 0.12 0.48	0.20 0.06 0.13 0.08 0.14 0.44	3.07 2.95 1.42 6.62 3.86 1.00
Number of scheduled hours ^a^ Less than 30 30 to less than 40 40 or more	[2] 0.32 0.39 0.29	[1,3,4] 0.25 0.31 0.44	[2] 0.39 0.43 0.18	[2] 0.43 0.31 0.26	23.32***
Telecommuting ^a^	[4] 0.50	0.58	0.68	[1] 0.67	8.56*
Job security ^b^	[2] 4.12(0.12)	[1,3,4] 4.90(0.08)	[2] 4.03(0.26)	[2] 4.08(0.14)	47.13***
Financial strain ^b^	[2,4] 2.27(0.08)	[1,3,4] 1.80(0.06)	[2] 2.47(0.15)	[1,2] 2.68(0.09)	82.29***
*Pandemic-related Factors*					
Perceived vulnerability to COVID-19 ^b^	[2,4] 4.36(0.06)	[1,4] 4.16(0.04)	4.31(0.12)	[1,2] 4.53(0.06)	26.80***
Social distancing ^a^	0.89	0.88	0.82	0.86	1.33

*Note*. ^a^ The DCAT function in MPlus was used to estimate trajectory membership. ^b^ The BCH function in MPlus was used to estimate trajectory membership. Trajectory numbers in square brackets (“[ ]”) indicate which Trajectory(ies), if applicable, the focal trajectory is significantly different from. χ^2^ = Chi-squared test. *** *p* ≤ .001, ** *p* ≤ .01, * *p* ≤ .05

#### Flourishing Model

The flourishing model (see Fig. 2b) contained three latent trajectories. Trajectory 1 (36.57% of the sample) had moderately high flourishing at study onset (4.82), which decreased linearly but decelerated (i.e., the pace of the decrease got smaller as time passed) to 4.64 at Time 2. Trajectory 2 had the most workers (56.17%). These workers had the highest flourishing; they had a high flourishing score at study onset (6.01), which decreased – although not significantly – to 5.95 at Time 2. Thus, Trajectory 2 was the best trajectory of the three. Trajectory 3 had the least workers (7.25%). These workers had the lowest flourishing; they had a moderately low flourishing score at study onset (3.18), which decreased – although not significantly – to 3.00 at Time 2. Thus, Trajectory 3 was the worst trajectory of the three.

#### Membership to Latent Flourishing Trajectories

As seen in Table 8, Trajectory 1 included a larger proportion of separated and divorced workers; however, the proportion was the same in Trajectory 3. There was also a larger proportion of workers scheduled to work less than 30 h per week in Trajectory 1. A larger proportion of workers who identified as men, had no disabilities, were married, worked full-time, or scheduled 40 h or more a week were in Trajectory 2. Workers in Trajectory 2 had the highest mean in age, trait resilience, positive family functioning, and sense of job security. Trajectory 2 had a larger proportion of Qualtrics workers. The demographic predictors of Trajectory 2 mirrored the sociodemographic breakdown of our Qualtrics subsample. A larger proportion of workers who identified as women or had one or more disabilities were found in Trajectory 3. Single workers, as well as workers in a common law relationship, were also more likely to be in Trajectory 3. Trajectory 3 had more workers characterized by one of many employment statuses (part-time, laid off, “other” unemployment), as well as workers scheduled to work 30 to less than 40 h a week. Workers in Trajectory 3 had the highest mean in financial strain and perceived vulnerability to COVID-19. Trajectory 3 had a larger proportion of social media workers. Most demographic predictors of Trajectory 3 mirrored the sociodemographic breakdown of our social media subsample.

**Table 8:** Association of Risk and Resilience Factors with Flourishing Trajectories

	Trajectory 1 Probability^a^or M (S.E.)^b^	Trajectory 2 Probability^a^or M (S.E.)^b^	Trajectory 3 Probability^a^or M (S.E.)^b^	χ^2^
Source Social media Qualtrics	[2] 0.55 0.45	[1,3] 0.29 0.71	[2] 0.68 0.32	43.82***
*Self-related Factors*				
Gender ^a^	[2]	[1,3]	[2]	24.99***
Men Women	0.27 0.74	0.42 0.58	0.15 0.85	
Age ^b^	[2] 38.69(0.85)	[1,3] 43.88(0.73)	[2] 37.68(1.81)	24.28***
Racial minority ^a^	0.10	0.13	0.11	1.15
Immigrant ^a^	0.15	0.19	0.15	1.01
Has one or more disabilities ^a^	[2,3] 0.19	[1,3] 0.09	[1,2] 0.39	20.24***
Trait resilience ^b^	[2,3] 2.90(0.06)	[1,3] 3.77(0.05)	[1,2] 2.33(0.14)	180.25***
*Social-related Factors*				
Marital status ^a^	[3]	[3]	[1,2]	19.89**
Single Common law Married Separated/Divorced	0.35 0.15 0.42 0.09	0.27 0.15 0.50 0.07	0.50 0.23 0.19 0.09	
Family functioning ^b^	[2,3] 2.32(0.04)	[1,3] 2.66(0.03)	[1,2] 1.93(0.09)	92.42***
Children at home ^a^	0.29	0.28	0.21	1.27
*Workplace-related Factors*				
Employment status ^a^		[3]	[2]	13.33*
Employed full-time Employed part-time Laid off due to COVID-19 Other unemployed (e.g., not employed but looking)	0.67 0.09 0.21 0.03	0.77 0.07 0.15 0.01	0.54 0.12 0.25 0.09	
Work industry ^a^				
Education Food Healthcare Manufacturing Services Other (e.g., automotive)	0.17 0.07 0.14 0.05 0.17 0.47	0.13 0.06 0.17 0.08 0.11 0.50	0.10 0.09 0.23 0.07 0.19 0.39	1.72 0.29 1.73 1.42 3.92 2.05
Number of scheduled hours ^a^	[2]	[1,3]	[2]	13.67**
Less than 30 30 to less than 40 40 or more	0.39 0.32 0.28	0.26 0.35 0.39	0.35 0.44 0.21	
Telecommuting ^a^	0.39	0.45	0.36	3.01
Job security ^b^	[2] 4.04(0.10)	[1,3] 4.80(0.08)	[2] 3.78(0.22)	44.46***
Financial strain ^b^	[2,3] 2.37(0.07)	[1,3] 1.93(0.05)	[1,2] 2.89(0.16)	51.00***
*Pandemic-related Factors*				
Perceived vulnerability to COVID-19 ^b^	[3] 4.33(0.05)	[3] 4.25(0.04)	[1,2] 4.54(0.09)	8.82 **
Social distancing ^a^	0.84	0.90	0.86	4.48

*Note*. ^a^ The DCAT function in MPlus was used to estimate trajectory membership. ^b^ The BCH function in MPlus was used to estimate trajectory membership. Trajectory numbers in square brackets (“[ ]”) indicate which Trajectory(ies), if applicable, the focal trajectory is significantly different from. χ^2^ = Chi-squared test. *** *p* ≤ .001, ** *p* ≤ .01, * *p* ≤ .05

#### Presenteeism Model

The presenteeism model (see Fig. 2c) contained five latent trajectories. Trajectory 1 (25.99% of the sample) had a moderately low presenteeism score at study onset (2.16), exponentially decreasing to 2.12 at Time 2. Trajectory 2 (5.02%) had a moderate presenteeism score at study onset (3.31), which decreased linearly and decelerated to 1.73 at Time 2. Trajectory 3 (16.49%) had a moderate presenteeism score at study onset (3.11), linearly increasing but decelerating to 2.93 at Time 2. Trajectory 4 contained the most workers (50.90%). These workers had the lowest presenteeism scores; they had a low presenteeism score at study onset (1.37), which decreased exponentially to 1.33 at Time 2. Thus, Trajectory 4 was the best trajectory of the five. Trajectory 5 contained the least workers (1.61%). These workers had the highest presenteeism scores; they had a high presenteeism score at study onset (4.66), which decreased – although not significantly – to 4.28 at Time 2. Thus, Trajectory 5 was the worst trajectory of the five.

#### Membership to Latent Presenteeism Trajectories

As seen in Table 9, Trajectory 1 had the largest proportion of social media workers; however, the proportion of social media workers was the same in Trajectories 3 and 5. Trajectory 2 had the most workers without a disability. Workers in a common law relationship or who were separated or divorced also had the largest proportion in Trajectory 2. Trajectory 2 had the largest proportion of workers scheduled to work 30 to less than 40 h a week. Trajectory 3 included a larger proportion of workers with one or more disabilities. Healthcare workers also had the largest proportion in Trajectory 3. Trajectory 4 included a larger proportion of workers who identified as men, were married, worked full-time, or scheduled 40 h or more a week. Workers in Trajectory 4 had the highest mean in age, trait resilience, positive family functioning, and sense of job security. Trajectory 4 had a larger proportion of Qualtrics workers. The demographic predictors of Trajectory 4 mirrored the sociodemographic breakdown of our Qualtrics subsample. Trajectory 5 included a larger proportion of workers who identified as women. A larger proportion of workers who were single, working part-time, laid off due to COVID-19, unemployed for a reason other than COVID-19 (e.g., retirement), or scheduled to work less than 30 h a week were found in Trajectory 5. Workers in manufacturing had the largest proportion in Trajectory 5 than the other trajectories. Trajectory 5 had workers with the highest mean in financial strain and perceived vulnerability to COVID-19. Whereas several presenteeism trajectories equally represented our social media subsample, the demographic predictors of the same trajectories mostly mirrored the sociodemographic breakdown of the social media subsample.

**Table 9:** Association of Risk and Resilience Factors with Presenteeism Trajectories

	Trajectory 1 Probability^a^or M (S.E.)^b^	Trajectory 2 Probability^a^or M (S.E.)^b^	Trajectory 3 Probability^a^or M (S.E.)^b^	Trajectory 4 Probability^a^or M (S.E.)^b^	Trajectory 5 Probability^a^or M (S.E.)^b^	χ^2^
Source Social media Qualtrics	[4] 0.59 0.41	[4] 0.50 0.50	[4] 0.59 0.41	[1,2,3,5] 0.14 0.86	[4] 0.59 0.42	88.84***
*Self-related Factors*						
Gender ^a^ Men Women	[4] 0.25 0.76	[4] 0.26 0.75	[4] 0.22 0.78	[1,2,3,5] 0.51 0.49	[4] 0.17 0.83	33.94***
Age ^b^	[4,5] 38.20(1.15)	[4] 38.74(2.24)	[4] 37.42(1.22)	[1,2,3,5] 47.49(0.75)	[1,4] 30.17(4.01)	90.45***
Racial minority ^a^	0.11	0.09	0.13	0.12	0.12	0.25
Immigrant ^a^	0.15	0.02	0.20	0.21	0.32	8.03
Has one or more disabilities ^a^	[2,3] 0.16	[1,3,4] 0.00	[1,2,4] 0.33	[2,3] 0.07	0.31	86.01***
Trait resilience ^b^	[3,4,5] 3.11(0.08)	[4,5] 2.83(0.21)	[1,4,5] 2.77(0.11)	[1,2,3,5] 3.83(0.05)	[1,2,3,4] 1.86(0.23)	177.33***
*Social-related Factors*						
Marital status ^a^	[5]		[5]	[5]	[1,3,4]	117.42***
Single Common law Married Separated/Divorced	0.35 0.17 0.42 0.07	0.26 0.32 0.27 0.15	0.39 0.19 0.38 0.05	0.23 0.12 0.57 0.08	0.59 0.00 0.33 0.08	
Family functioning ^b^	[3,4] 2.46(0.05)	2.47(0.15)	[1,4] 2.28(0.07)	[1,3] 2.64(0.03)	2.31(0.18)	29.08***
Children at home ^a^	0.26	0.38	0.33	0.30	0.23	1.54
*Workplace-related Factors*						
Employment status ^a^	[5]			[5]	[1,4]	25.04*
Employed full-time Employed part-time Laid off due to COVID-19 Other unemployed (e.g., not employed but looking)	0.82 0.11 0.04 0.03	0.78 0.08 0.15 0.00	0.74 0.16 0.09 0.01	0.89 0.06 0.05 0.002	0.36 0.31 0.23 0.11	
Work industry ^a^						
Education Food Healthcare Manufacturing Services Other (e.g., automotive)	0.18 0.07 [5] 0.15 [2] 0.05 0.16 0.50	0.07 0.07 [5] 0.18 [1,3,4] 0.00 0.21 0.58	0.20 0.004 [5] 0.22 [2] 0.05 0.08 0.45	0.11 0.04 [5] 0.19 [2] 0.09 0.10 0.51	0.22 0.23 [1,2,3,4] 0.00 0.11 0.26 0.30	6.27 2.60 120.03*** 40.55*** 4.34 2.61
Number of scheduled hours ^a^			[4]	[3]		20.86**
Less than 30 30 to less than 40 40 or more	0.26 0.41 0.33	0.26 0.58 0.16	0.32 0.43 0.26	0.20 0.33 0.48	0.40 0.37 0.23	
Telecommuting ^a^	0.50	0.37	0.47	0.46	0.52	0.51
Job security ^b^	[2,4] 4.16(0.14)	[1,3] 4.80(0.24)	[2,4] 4.19(0.17)	[1,3,5] 4.88(0.08)	[4] 3.81(0.52)	28.65***
Financial strain ^b^	[3,4,5] 2.14(0.09)	[3,5] 1.82(0.17)	[1,2,4] 2.49(0.11)	[1,3,5] 1.84(0.06)	[1,2,4] 2.80(0.33)	36.55***
*Pandemic-related Factors*						
Perceived vulnerability to COVID-19 ^b^	[4] 4.43(0.07)	[4] 4.51(0.13)	4.34(0.09)	[1,2] 4.16(0.04)	4.53(0.21)	17.96***
Social distancing ^a^	0.84	0.94	0.87	0.87	0.89	1.55

*Note*. ^a^ The DCAT function in MPlus was used to estimate trajectory membership. ^b^ The BCH function in MPlus was used to estimate trajectory membership. Trajectory numbers in square brackets (“[ ]”) indicate which Trajectory(ies), if applicable, the focal trajectory is significantly different from. χ^2^ = Chi-squared test. *** *p* ≤ .001, ** *p* ≤ .01, * *p* ≤ .05

### Thriving at Work Model

The thriving at work model (see Fig. 2d) contained four latent trajectories. Trajectory 1 contained the most workers (38.27%). These workers had moderately high thriving at work at study onset (4.28), which decreased – although not significantly – to 4.12 at Time 2. Trajectory 2 had 37.95% of the sample. These workers had the highest thriving at work; they had a high thriving at work score at study onset (5.55), which decreased linearly but decelerated to 5.37 at Time 2. Thus, Trajectory 2 was the best trajectory of the four. Trajectory 3 contained the least workers (9.13%). These workers had the lowest thriving at work; they had a low thriving at work score at study onset (1.63), which decreased – although not significantly – to 1.43 at Time 2. Thus, Trajectory 3 was the worst trajectory of the four. Trajectory 4 (14.65% of the sample) had moderately low thriving at work at study onset (2.88), which decreased – although not significantly – to 2.86 at Time 2.

#### Membership to Latent Thriving at Work Trajectories

As seen in Table 10, Trajectory 1 included a larger proportion of workers who identified as men or had children at home. The proportion of men, however, was equal to that in Trajectory 2. Educational workers or workers scheduled 30 to less than 40 h a week also had a higher proportion in Trajectory 1. The proportion of those scheduled to work 30 to less than 40 h a week was equal in Trajectory 2. A larger proportion of workers who were immigrants, had no disabilities, or married were in Trajectory 2. The proportion of immigrants, however, was equal to that of Trajectory 4. Trajectory 2 had the largest proportion of workers employed full-time, in healthcare, scheduled 40 or more hours a week, or telecommuting. Workers in Trajectory 2 had the highest mean in age, trait resilience, positive family functioning, and sense of job security. Trajectory 2 had a larger proportion of Qualtrics workers. The demographic predictors of Trajectory 2 mostly mirrored the sociodemographic breakdown of our Qualtrics subsample. A larger proportion of workers who were born in Canada, or had no children at home were in Trajectory 3. Workers who had one or more disabilities, were single, or in a common law relationship were found in Trajectory 3. However, an equal proportion of workers with at least one disability was found in Trajectory 4. Trajectory 3 had a larger proportion of workers who were not telecommuting, were employed part-time, unemployed for a reason other than COVID-19 (e.g., retirement), or employed in the food or services industry. Trajectory 3 had workers with the highest mean in financial strain and perceived vulnerability to COVID-19. Trajectory 3 had a larger proportion of social media workers. The demographic predictors of Trajectory 3 mirrored the sociodemographic breakdown of our social media subsample. Trajectory 4 had the largest proportion of women, as well as separated and divorced workers. The largest proportion of workers laid off due to COVID-19 or in an “other” work industry (e.g., automotive) were found in Trajectory 4. Workers scheduled less than 30 h a week were found in Trajectory 4.

**Table 10:** Association of Risk and Resilience Factors with Thriving at Work Trajectories

	Trajectory 1 Probability^a^or M (S.E.)^b^	Trajectory 2 Probability^a^or M (S.E.)^b^	Trajectory 3 Probability^a^or M (S.E.)^b^	Trajectory 4 Probability^a^or M (S.E.)^b^	χ^2^
Source	[3]	[3]	[1,2]		14.17**
Social media Qualtrics	0.39 0.62	0.37 0.63	0.63 0.38	0.48 0.52	
*Self-related Factors*					
Gender ^a^	[3,4]	[3,4]	[1,2]	[1,2]	13.95**
Men Women	0.38 0.62	0.38 0.62	0.22 0.78	0.21 0.79	
Age ^b^	[3] 41.81(0.88)	[3,4] 43.68(0.89)	[1,2,4] 34.73(1.65)	[2,3] 40.00(1.51)	25.99***
Racial minority ^a^	0.13	0.11	0.06	0.12	2.60
Immigrant ^a^	[3] 0.16	[3] 0.20	[1,2,4] 0.07	[3] 0.20	8.52*
Has one or more disabilities ^a^	0.13	[4] 0.12	0.24	[2] 0.24	7.94*
Trait resilience ^b^	[2,3] 3.23(0.07)	[1,3,4] 3.71(0.06)	[1,2] 2.88(0.13)	[2] 3.03(0.12)	59.39***
*Social-related Factors*					
Marital status ^a^	[3,4]	[3,4]	[1,2]	[1,2]	43.53***
Single Common law Married Separated/Divorced	0.33 0.12 0.50 0.05	0.27 0.13 0.53 0.08	0.42 0.28 0.21 0.09	0.37 0.22 0.24 0.17	
Family functioning ^b^	[3,4] 2.54(0.04)	[3,4] 2.62(0.04)	[1,2] 2.11(0.09)	[1,2] 2.24(0.07)	47.68***
Children at home ^a^	[3] 0.32	[3] 0.29	[1,2] 0.13	0.24	11.74**
*Workplace-related Factors*					
Employment status ^a^	[2,3,4]	[1,3,4]	[1,2]	[1,2]	44.30***
Employed full-time Employed part-time Laid off due to COVID-19 Other unemployed (e.g., not employed but looking)	0.77 0.09 0.11 0.03	0.81 0.04 0.15 0.00	0.49 0.17 0.27 0.07	0.58 0.11 0.31 0.00	
Work industry ^a^					
Education Food Healthcare Manufacturing Services Other (e.g., automotive)	[3,4] 0.19 0.07 [3] 0.15 0.05 0.16 [4] 0.41	0.13 [3]0.03 [3,4] 0.23 0.08 [3] 0.10 0.51	[1] 0.08 [2]0.17 [1,2,4] 0.02 0.06 [2,4] 0.30 0.47	[1] 0.08 0.10 [2,3] 0.12 0.10 [3] 0.08 [1] 0.62	8.43* 12.36** 32.96*** 1.19 9.00* 8.32*
Number of scheduled hours ^a^		[4]		[2]	16.02**
Less than 30 30 to less than 40 40 or more	0.29 0.37 0.34	0.23 0.37 0.40	0.40 0.29 0.31	0.46 0.28 0.25	
Telecommuting ^a^	[3] 0.44	[3] 0.48	[1,2,4] 0.18	[3] 0.40	22.56***
Job security ^b^	[3,4] 4.51(0.10)	[3,4] 4.76(0.10)	[1,2] 3.50(0.20)	[1,2] 4.01(0.16)	46.71***
Financial strain ^b^	[2,3,4] 2.14(0.07)	[1,3,4] 1.93(0.06)	[1,2] 2.60(0.15)	[1,2] 2.45(0.13)	30.87***
*Pandemic-related Factors*					
Perceived vulnerability to COVID-19 ^b^	4.30(0.05)	[3] 4.22(0.05)	[2] 4.52(0.10)	4.36(0.08)	8.09*
Social distancing ^a^	0.87	0.88	0.83	0.90	1.00

*Note*. ^a^ The DCAT function in MPlus was used to estimate trajectory membership. ^b^ The BCH function in MPlus was used to estimate trajectory membership. Trajectory numbers in square brackets (“[ ]”) indicate which Trajectory(ies), if applicable, the focal trajectory is significantly different from. χ^2^ = Chi-squared test. *** *p* ≤ .001, ** *p* ≤ .01, * *p* ≤ .05

## Discussion

This study explored well-being trajectories Canadian workers were experiencing across multiple well-being indicators during the first few months of the COVID-19 pandemic. We first hypothesized that intragroup differences would be present in levels of well-being at study onset (H1). Support for H1 was found in that intragroup differences were present in scores for each well-being indicator at study onset. Our second hypothesis was that different trajectories would emerge for each well-being indicator, such that some workers’ scores would get better, some workers’ scores would get worse, and some workers’ scores would remain the same through time (H2). Some support was found for H2 as some workers’ scores got better, whereas some worsened or stagnated over time depending on the well-being indicator explored. Lastly, we hypothesized that multiple factors found at different ecological levels (self, social, workplace, pandemic) would predict well-being trajectories’ membership. Some support was found in that factors at different ecological levels (self, social, workplace, pandemic) predicted being a member of different well-being trajectories.

### Understanding Workers’ Holistic Well-Being

Exploration of a relatively comprehensive set of workers’ well-being during COVID-19 led to three intriguing findings. First, there are more latent trajectories for negative (vs. positive) well-being in workers’ general lives and work. This is like our cross-sectional study, where there was more variability in the severity of suffering workers experienced than the prospering they experienced (Pacheco et al., 2024). Second, across all the trajectories in the well-being models, negative well-being improves over time or stays stagnant, whereas positive well-being worsens or stays stagnant. Uncertainty regarding contracting or dying from the pathogen and becoming unemployed could be a reason why people suffered at the psychological level during COVID-19 (Wu et al., 2022). The pandemic’s beginning could be characterized by heightened uncertainty, thus leading to higher negative well-being at pandemic onset. Here, workers’ negative well-being at study onset could have been affected to different degrees, thus resulting in different intercepts in the trajectories. Negative well-being could have improved over time as pandemic conditions settled and new ways of living became normal. Pandemic contexts, however, are not circumstances that support positive well-being (e.g., Flaskerud, 2022). This may explain why workers experienced deterioration or stagnation in their positive well-being over time. Third, there was more stability in both positive (vs. negative) well-being indicators’ trajectories. The negative well-being indicators that we explored, in contrast, were hedonic. This distinctive nature of the negative well-being indicators may explain why they changed more than the positive well-being indicators over the three timepoints. As indicators of positive well-being, a sense of flourishing and thriving at work reflects eudaimonic well-being, characterized by personal and social skills and abilities (Ryff, 2018), may have a lower change intensity than hedonic forms of negative well-being (e.g., distress). This reflects similar changes in longitudinal tourism-based research on hedonic and eudaimonic well-being following holidays, in which eudaimonic well-being had a lower change intensity than hedonic well-being (e.g., Su et al., 2020; Yu et al., 2021). Thus, more time may be required to see a change in these well-being indicators.

### Factors Affecting Workers’ Well-Being

**Fig. 3:**
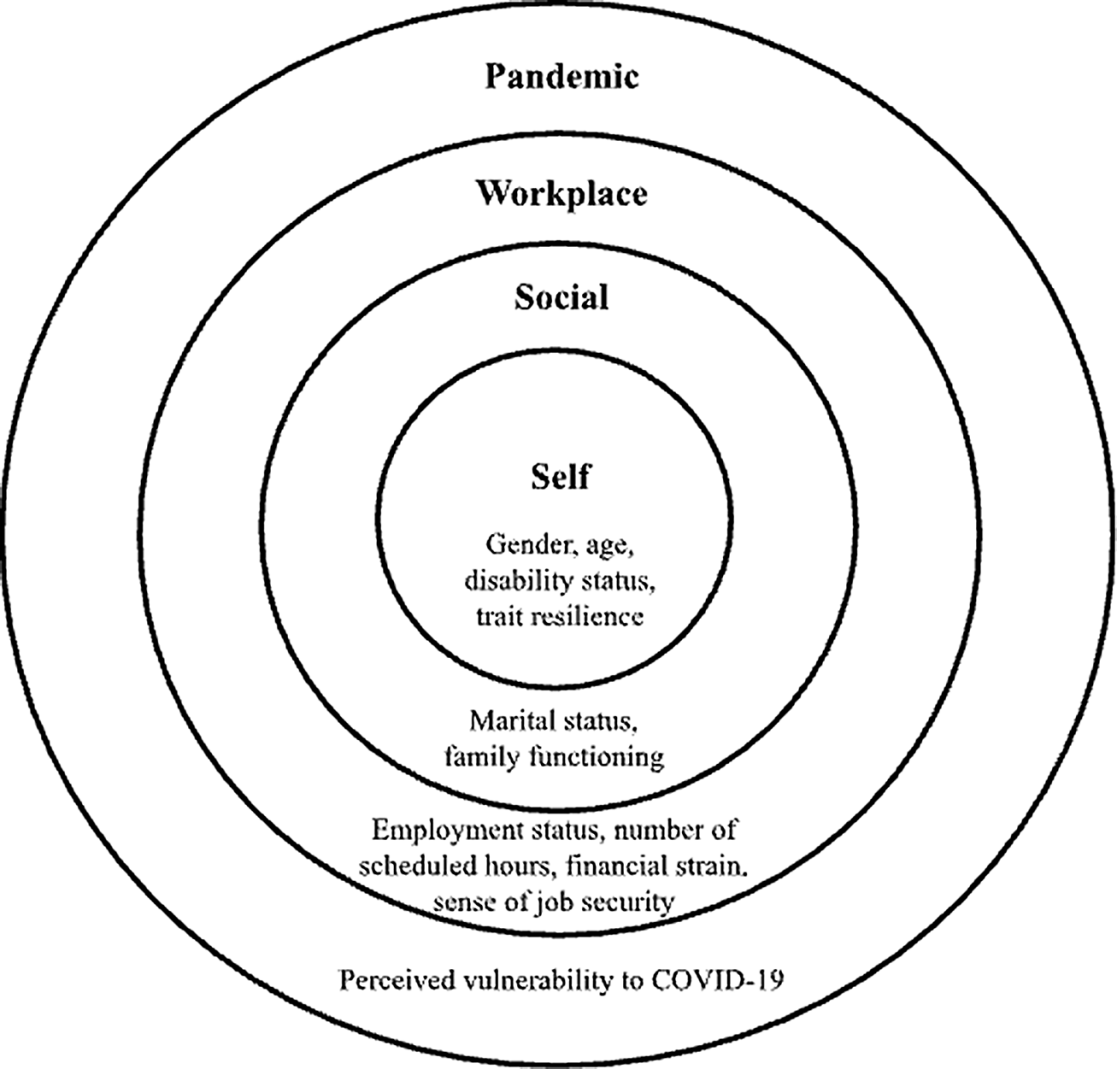
The Ecological Nesting of the Significant Predictors of Well-being Trajectory Membership

Factors nested in the self-, social-, workplace-, and pandemic-related ecological levels significantly predicted membership to well-being trajectories across the well-being indicators (see Fig. 3). This section discusses these results as they relate to previous research and provides recommendations for diverse stakeholders.

#### Self-related Factors

Identifying as a woman, being younger, and having less trait resilience were associated with membership to a relatively worse well-being trajectory. These findings are similar to conclusions made in previous literature and our cross-sectional study, in which women, being younger, and having lower trait resilience were related to worse well-being (e.g., Carney et al., 2021; Hansen & Blekesaune, 2022; Hu et al., 2015) and less odds of experiencing a prospering reality (Pacheco et al., 2024). To support these workers’ well-being, mental health organizations could, for example, develop programming unique to these workers or make them more (financially) accessible. Additionally, and like Chin et al.’s (2023) perspective on race-based trauma, findings regarding the belonging of diverse minority groups (e.g., race as a predictor in the distress model) in more prospering trajectories are consistent with previous work suggesting that encountering discrimination may promote some group members’ resilient responses to future adversity through post-traumatic growth processes (e.g., Grier-Reed et al., 2023; Seery et al., 2010).

#### Social-related Factors

Although not hypothesized in the current study due to contrasting results in our cross-sectional study (Pacheco et al., 2024), marital status significantly predicted trajectory membership in three of the four well-being models. In these models, and like the cross-sectional study, however, married workers were consistent members of the best trajectories in these models. Single workers were members of the worst trajectories. These results are also consistent with other previous research indicating benefits (e.g., lower mortality) of being married versus single (e.g., Waite, 1995).

Like other previous research (e.g., Grevenstein et al., 2019), more positive family functioning was associated with membership to relatively better trajectories. Grevenstein et al. (2019) found that better family relationships were associated with reduced distress and increased life satisfaction, for example. Employers could help promote family functioning by fostering a workplace culture founded on work-life balance. Adopting safeguarding against work intensification (i.e., preventing demanded heightened focus and engagement in workers) can be used by employers to promote work-life balance (Kossek et al., 2014). Moreover, and as predicted, having a child at home significantly predicted trajectory membership for thriving at work. Workers with children had moderately high scores of thriving at work; workers with no children were members of the worst thriving at work trajectory characterized by very low scores at each timepoint. Further research is needed to explore this overlap of well-being domains to disentangle why having children at home is associated with more prospering at work.

#### Workplace-related Factors

Some predictors provide insight into the harmful nature of two dimensions of precarious employment: employment insecurity and income inadequacy (Kreshpaj et al., 2020). Reflecting the employment insecurity dimension, full-time workers were consistently members of the best trajectories in each well-being model. The other employment statuses predicted membership to relatively worse trajectories when compared to being employed full-time. In a similar vein, except flourishing, workers with more scheduled hours were members of a relatively better trajectory. These findings are reminiscent of research showing that being employed full-time (vs. unemployed/part-time workers) is associated with psychological and physical benefits (e.g., lower stress and depressive symptoms, less cigarette and alcohol use) (Rosenthal et al., 2012). Similarly, workers with the highest mean sense of job security scores were members of the best trajectories across the well-being indicators. Regarding the second dimension of precarious employment, income inadequacy, workers with the highest financial strain were consistently in the worst well-being trajectories. Enactment of the Canada Emergency Response Benefit Act on March 25th, 2020 allowed eligible workers to collect an income from the government (Government of Canada, 2023). Although the act aided workers facing financial strain, it reflects a secondary prevention activity (i.e., an activity focused on intervening in the context of an emerging issue) (Government of Canada, 2013). From a public health approach, enacting a primary prevention approach (i.e., activities preventing and focusing on reducing factors leading to health problems) would be more optimal. Developing policies that aid workers *if* a public health crisis were to occur would prevent negative outcomes (e.g., lack of resources and increased mental suffering) from occurring.

Telecommuting status was not hypothesized to be predictive of the well-being trajectories, because it was not predictive of the cross-sectional profiles in our previous publication (Pacheco et al., 2024). Here, however, telecommuting status did significantly predict trajectory membership for distress and thriving at work. Telecommuters were more likely to be in a trajectory characterized by high-onset distress that decreased greatly longitudinally. Telecommuters were more likely to be in Trajectory 2 for thriving at work, characterized by high thriving at work across time. These findings highlight a juxtaposition: workers can have high distress while telecommuting but also experience high thriving at work. The high distress at study onset could be explained by considering findings from Otsuka et al.’s (2021) study, which found that those who did not want to telecommute during the COVID-19 pandemic had higher odds (1.87) of experiencing distress. It is possible that several Canadian workers who began telecommuting during COVID-19 may not have wanted to do so initially. Workers, over time, may have adapted to the shift in work modes. Research, however, is needed to explore how these well-being indicators can co-occur.

#### Pandemic-related Factors

Higher scores in perceived vulnerability to COVID-19 at the study onset consistently predicted membership in the worst well-being trajectories. Findings associated with this factor are reminiscent of previous research showing that increased risk or exposure to a pathogen is related to poorer well-being (e.g., Wu et al., 2009). Pacheco et al. (2024), citing results from Falco et al. (2021), suggest that implementing and using safety systems, communication, and (participating in) decision-making may be a cost-effective way of reducing perceived risk of work-related infection and improving workers’ well-being during pandemics and epidemics. The predictive power of workers’ perceived vulnerability to COVID-19 suggests the importance of prioritizing the implementation of these buffers. Earlier implementation could reduce the likelihood of workers experiencing negative trajectories.

Social distancing, interestingly, did not predict trajectory membership. As the continual practice of social distancing may be associated with several negative outcomes (e.g., Su et al., 2023), the lack of predictive power may be because workers had not been socially distancing long enough to experience a clear deleterious effect.

## Strengths and Limitations

In this study, theories related to industrial-organizational psychology, developmental and community psychology, positive psychology, and public health were married using the third wave of positive psychology. Related to Wissing’s (2022) position on understanding COVID-19 best, the multidisciplinary perspective adopted aided in understanding the well-being trajectories found across positive and negative well-being indicators and which workers were most likely members of each trajectory. LTA allowed us to identify that workers were either stagnant or improving over time regarding their distress and presenteeism and stagnant or worsening concerning their flourishing and thriving at work.

The first limitation associated with this study is that our sample is not fully generalizable as we were not able to reach a larger representation from some groups (e.g., workers with a disability, racialized workers). Low subsamples in some demographic groups could have affected the estimation process. Moreover, we removed participants who had less than two valid timepoints from these analyses. These workers, however, had significantly worse well-being. This could have affected (a) the number of trajectories found, and (b) the shape of the trajectories. A second limitation is that our study only had three timepoints at the pandemic onset. As the pandemic began with several stressors (e.g., social distancing measures, workplace closures), these findings represent a turbulent period during COVID-19. It is plausible that trajectories would show improved well-being through time. Thus, these results should not be considered representative of workers’ well-being after May 2020. Third, related to limitations with person-centered analyses more generally, LTA did not allow us to explore the prospective association between the study variables and well-being levels across the general sample through time. Lastly, we selected several indicators of workers’ general and work-related well-being, including both positive and negative aspects, to test our hypotheses. Exploration of other well-being constructs could reveal different trajectories and/or slopes. Future research should explore other well-being constructs during such crises.

## Conclusion

No singular experience is generalizable of (Canadian) workers’ well-being during the onset of the COVID-19 pandemic. Many workers appear to be members of trajectories characterized by lower negative and higher positive well-being, indicating that many Canadian workers adapted well to COVID-19-related circumstances. Interestingly, Canadian workers’ distress and presenteeism scores generally improved or stayed stagnant. In contrast, their sense of flourishing and thriving at work generally worsened or remained stagnant. Illustrating the benefit of using Ecological Systems Theory, several factors at self-, social-, workplace-, and pandemic-related ecological levels were found to predict which workers were likely to experience more or less prospering or suffering well-being trajectories. Interventions that can change the course of suffering workers’ well-being while supporting the well-being of those prospering should be designed by policymakers and practitioner and implemented as primary prevention means to help deal with potential future pandemics and similar crises effectively.

## Footnotes

1 One hundred and eighty-four participants joined at Times 1 or 2. As participants who completed Time 0 were re-contacted to participate in later surveys, there should only have been missing data at this timepoint if it was because participants failed multiple attention checks. However, it seemed that a few participants likely shared the survey with other workers who did not complete Time 0 and who would have otherwise had no access to the later surveys. The 184 workers who joined the study at Time 1 or 2 indicated they had work experience, met the other inclusion criteria, and correctly answered more than 50% of the attention checks. With MPlus’ ability to handle their missing data at Time 0 (Muthén & Muthén, 1998–2017), we retained these workers’ data for the first objective. These data were not used for the second objective as Time 0 data were used as predictors of the well-being trajectories.
